# Biliobronchial Fistula after Liver Surgery for Giant Hydatid Cyst

**DOI:** 10.1155/2011/347654

**Published:** 2011-09-22

**Authors:** Carmelo Loinaz, Teresa Hernández, Mercedes Mitjavila, Jaime Martín, Federico Ochando, Maria Lucia Madariaga, Beatriz Fernández, Pilar Hernández, José Rueda, María Ramos, Pedro Jiménez, Peter Vorwald, José María Fernández, Antonio Quintáns

**Affiliations:** ^1^Unidad de Cirugía General, Fundación Hospital Alcorcón, Budapest 1, Alcorcón, 28922 Madrid, Spain; ^2^Area de Radiología y Medicina Nuclear, Fundación Hospital Alcorcón, 28922 Madrid, Spain; ^3^Department of Surgery, Massachusetts General Hospital, Boston, MA 02114, USA

## Abstract

*Background*. Biliobronchial fistula (BBF) is a rare complication in the natural history of liver hydatid disease by *Echinococcus granulosus*. We present a case of BBF after resection of a giant liver hydatid cyst in a 72-year-old woman. *Case Report*. A total cystpericystectomy was done, leaving the left lateral section of the liver that was fixed to the diaphragm. Postoperatively, the patient developed obstructive jaundice. An ERCP showed an obstruction at the junction of the left biliary duct and the main biliary duct and contrast leak. At reoperation, the main duct was ischemic, likely due to torsion along its longitudinal axis. A hepatotomy was done at the hilar plate, and the biliary duct was dissected and anastomosed to a Roux-en-Y jejunal loop. She was discharged without complications. Five months later, the patient developed cholangitis and was successfully treated with antibiotics. However, she suffered repeated respiratory infections, and four months later she was admitted to the hospital with fever, cough, bilioptysis, and right lower lobe pneumonia. The diagnosis of BBF was confirmed with ^99m^Tc Mebrofenin scintigraphy. At transhepatic cholangiography, bile duct dilation was seen, with a biliothoracic leak. She underwent dilatation of cholangiojejunostomy stricture with placement of an external-internal catheter. The catheter was removed 3.5 months later, and two years later the patient remains in very good condition. *Conclusion*. An indirect treatment of the BBF by percutaneous transhepatic dilation of the biliary stenosis avoided a more invasive treatment, with satisfactory outcome.

## 1. Introduction

Bronchobiliary fistula (BBF) is defined as an abnormal communication between the biliary tree and the bronchial tract. It is a rare complication that may appear in the natural history of liver hydatid disease or after its surgical treatment [1, 2], trauma [3], congenital malformation [4–6], tumor [7], surgery [8–10], biliary lithiasis [11], hepatic abscess [12], liver transplantation [13], or even radiofrequency treatment [14, 15].

The most prominent sign is bilioptysis or the presence of bile in the sputum. Other possible symptoms and signs are cholangitis, jaundice, and cutaneous fistula [12]. The first case of BBF was described by Peacock in 1850 [16] in a patient with a hydatid cyst.

We present a case of BBF that appeared after the surgical treatment of a giant liver hydatid cyst.

## 2. Case Report

**Figure 1:**
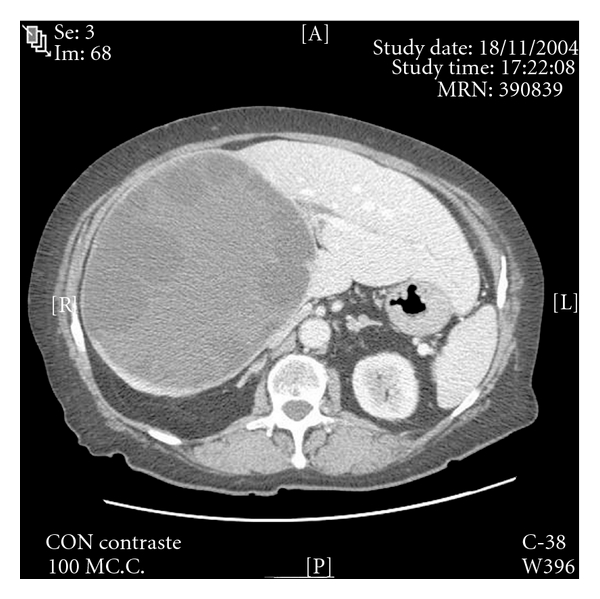
Preoperative abdominal CT. Massive liver hydatid cyst replacing segments IV to VIII.

A 72-year-old woman with abdominal complaints for several months was found to have a large right upper quadrant cystic mass (16 × 17 × 18 cm), consistent with a multivesicular liver hydatid cyst that replaced the right lobe of the liver, with compensatory hypertrophy of the left lateral segment (LLS) (Figure 1). After discussing the high risk of postoperative complications, the patient agreed to undergo surgery. We performed a total open cyst pericystectomy; after detaching the right lobe ligaments, we opened the cyst, extracted the fluid, and instilled hypertonic saline. The right lobe elements were ligated, and the entire cyst with a margin of pericystic inflammation was resected. The LLS was sutured to the diaphragm. 

**Figure 2:**
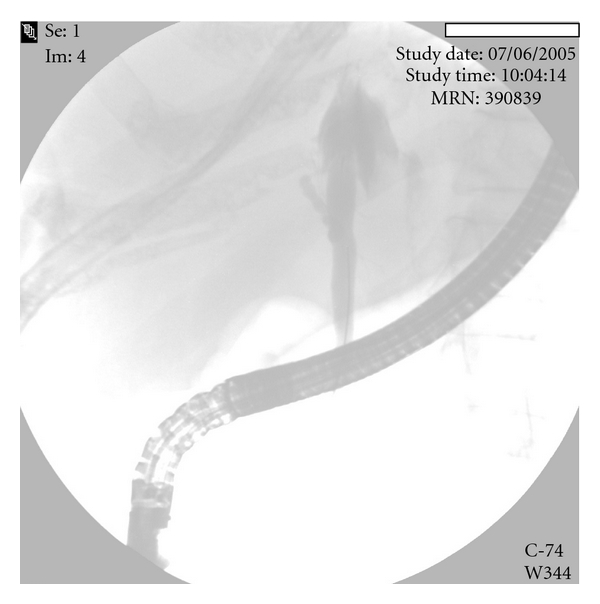
ERCP showing a stop at the level of the biliary confluence and a contrast leak.

Postoperatively, the patient developed progressive jaundice. She was afebrile and had otherwise normal liver function. Ultrasound and CT were notable for intrahepatic biliary dilation. ERCP showed an obstruction at the level of the biliary confluence and a contrast leak (Figure 2). The patient was taken back to surgery for exploration where we found a fibrotic and ischemic main bile duct, likely due to torsion along its longitudinal axis. We dissected the bile duct until the liver parenchyma, but there was not viable tissue. A hepatotomy was performed at the hilar plate and a bile duct of about 1 cm in diameter was dissected and opened longitudinally. A Roux-en-Y jejunal loop was prepared. A side-to-side biliary jejunal anastomosis was performed with interrupted 6–0 PDS sutures. The patient recovered without further complications, and she was discharged one week later.

Five months after the initial surgery, the patient was admitted to the hospital with ascending cholangitis. She was successfully treated with antibiotics and discharged.

**Figure 3:**
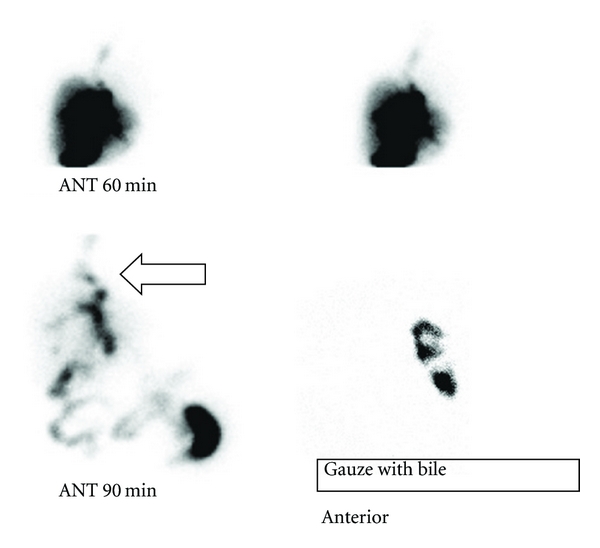
^99m^Tc Mebrofenin scintigraphy: a fistulous tract is seen connecting the liver and the lower right lobe (arrow). A gauze with the patient sputum had enough radioisotopes to be detected by the gamma camera (right, bottom). ANT: anterior view.

However, the patient suffered repeated respiratory infections in the outpatient setting. Nine months after the original operation, the patient presented to the hospital with fever and productive cough. She did not have jaundice. On physical examination, the patient seemed uncomfortable and expectorated bile-stained sputum several times. Chest plain film showed right lower lobe opacity consistent with right lower lobe pneumonia. Because of her persistent bilioptysis, she underwent ^99m^Tc Mebrofenin scan (Bridatec, Amersham Health, Gipharma, Saluggia, Italy) which confirmed the diagnosis of biliobronchial fistula (BBF). A fistulous tract connected the liver and the right lower lobe; the patient's sputum contained enough radioisotopes to be detected by the gamma camera (Figure 3).

**Figure 4:**
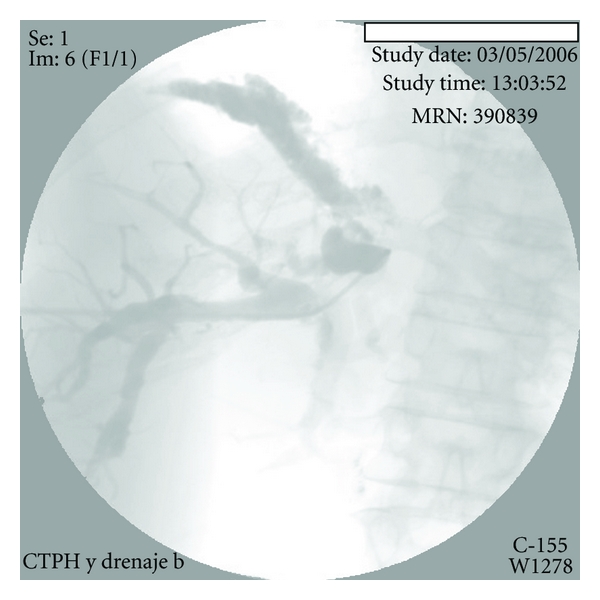
Percutaneous transhepatic cholangiography. Bile duct dilation is seen, with a biliothoracic leak (BBF).

Given the postsurgical anatomy, the patient underwent percutaneous transhepatic cholangiography which was notable for bile duct dilatation, stenosis of the biliojejunal anastomosis, and biliothoracic leak (BBF) (Figure 4). The anastomotic stricture was dilated, and an external-internal catheter was left in place. There was immediate cessation of bilioptysis and marked improvement of the patient's condition. 

Follow-up studies showed good contrast passage, without fistula or stenosis. The catheter was removed after 3.5 months. Two years later, the patient is in very good condition, asymptomatic, and enjoying good quality of life.

## 3. Discussion

The reason BBFs develop is not completely known. In some cases, inflammation by bile from the intra-abdominal compartment could permeate the diaphragm [8]. In our case, anastomotic stricture at the cholangiojejunostomy increased pressure in the biliary system, resulting in a pro-inflammatory collection of bile that extended through the diaphragm to communicate with pulmonary tissue. 

BBF is not easily diagnosed. Bile in the sputum is pathognomonic of BBF, but very often the patients have respiratory and/or biliary symptoms for weeks or months before this complication is diagnosed [2].

 In our case, BBF was not suspected until the patient started to have bilioptysis, and the suspicion was confirmed with a Mebrofenin scan, showing the fistulous tract and an isotope-containing sputum. The use of IDA scan was suggested as early as 1983 for this indication [17]. In the case of Tran et al. [15], the IDA scan showed not only tracer activity in the right lung field but also in the trachea and the oral cavity.

CT is widely used, but it rarely serves to visualize the fistula itself [8]. A biloma can be found, as a subphrenic collection, sometimes associated to a pleural effusion. In other instances, biliary dilatation can be seen, as in our case.

MRCP has been useful in some reports [8, 10]. It has limitations in the visualization of nondistended ducts and when there is pneumobilia, as is expected after cholangio-jejunostomy.

Treatment of BBF has traditionally been surgical repair [12, 18]. In 1990, Brem et al. reported the first case of successful endoscopic treatment of BBF, in a patient with biliary lithiasis [19]. Possible techniques are sphincterotomy alone [19, 20], stenting [21], or both [7]. Octreotide can be used as an adjunct to endoscopic treatment [22, 23].

In our case, as the patient had a previous biliary surgery with cholangiojejunostomy, we decided to use percutaneous transhepatic cholangiography to assess the biliary tree. After the diagnosis of biliary stenosis and BBF became apparent, dilatation of the stenotic site was performed, and an internal-external catheter was left in place [24], with the satisfactory result. This less invasive approach should be considered before reoperation.
